# Infiltration of Mast Cells in Scalp Biopsies of Patients with Alopcia Areata or Androgenic Alopecia Versus Healthy Individuals: A Case Control Study

**DOI:** 10.31661/gmj.v9i0.1962

**Published:** 2020-12-29

**Authors:** Soheila Nasiri, Alireza Salehi, Azadeh Rakhshan

**Affiliations:** ^1^Skin Research Center, Shahid Beheshti University of Medical Sciences, Tehran, Iran; ^2^Department of Pathology, Shohada-e-Tajrish Educational Hospital, School of Medicine, Shahid Beheshti University of Medical Sciences, Tehran, Iran

**Keywords:** Alopecia Areata, Androgenic Alopecia, Mast Cell, Inflammation

## Abstract

**Background:**

Alopecia areata (AA) and androgenic alopecia (AGA) are of the most common types of alopecias. Recently, the role of mastcells in inflammatory diseases has become the focus of many studies. However, few studies have been conducted on their role in AA and AGA. Therefore, our study aimed to quantitatively evaluate the presence of mastcells in the AA and AGA specimens.

**Materials and Methods:**

Three groups of AA, AGA, and healthy control were studied (each group with 20 subjects). Patients were randomly selected from those referred to the dermatology clinics of Shahid Beheshti University. Specimens were obtained from the scalp, and perifollicular and perivascular areas were investigated.

**Results:**

Significantly higher perifollicular and perivascular mastcell counts were seen in both AGA and AA groups as compared to healthy control (P<0.001 for both). Moreover, AA patients had more frequent perivascular mastcells than the AGA group (P=0.042). Among patients aged <40 years, perifollicular and perivascular mastcell counts were not significantly different among three groups; however, subjects over 40 years of age in both groups had significantly more perifollicular and perivascular mastcells than healthy participants. There was a significant positive correlation between disease severity and mast cell counts in both perifollicular and perivascular areas in AA patients (P=0.001 for both).

**Conclusion:**

There is a significantly increased infiltration of mastcells in AA and AGA patients, and this increase is age and severity dependent. Moreover, the increase in mastcell proliferation is more dominant in AA patients.

## Introduction

Primary alopecia of the scalp is categorized into two major types of scarring alopecias such as lichen planopilaris (follicular form of lichen planus) and frontal fibrosing alopecia and non-scarring alopecia such as androgenic alopecia (AGA) and alopecia areata (AA) [1,2]. Although the reported prevalence of alopecia varies greatly among different studies, the global lifetime incidence of AA is estimated to be about 2 percent [3]. Correspondingly, approximately 2 percent of outpatients’ visits are due to AA in the United States and the United Kingdom [4]. AGA is also a type of persistent, dynamic hair loss that affects about 90 percent of white males and 50 percent of white females in their lifetime [5]. Histopathologic features of AA are namely perifollicular lymphocyte infiltrations around the anagen hair follicles, which include both CD4+ and CD8+ cells. Other features include observation of eosinophils, lymphocytes, and melanin in fibrous tracts, pigment casts within follicular canals as well as catagen, and miniaturized follicles [6]. Moreover, perifollicular inflammation with subsequent perifollicular fibrosis is the most constant histopathologic findings in AGA [7]. Both human and animal studies have shown that mast cells have essential role in the onset and progression of autoimmune and inflammatory disorders [8,9]. For instance, in rheumatoid arthritis disease, a 6 to 25-fold increase in mast cell count has been reported in the affected joints compared to healthy joints [5]. Nevertheless, little is known about the role of mast cells in the pathophysiology of both AA and AGA. Considering the high value of investigating the cellular basis of diseases in the development of novel therapeutic modalities, we conducted this study to quantitatively evaluate the presence of mast cells in the AA and AGA specimens.

## Materials and Methods

The present study had a case-control design in which patients with AA and AGA were compared with healthy controls. The study was conducted following the declaration of Helsinki, and we obtained an informed consent from all participants after an adequate explanation of the study. The protocol of the study was approved by the medical ethics committee of Shahid Beheshti University of Medical Sciences, Iran (IR.SBMU.SRC.REC.1397.010). Patients were randomly selected (systematic sampling) from those referred to the dermatology clinics of Shahid Beheshti University of Medical Sciences between February 2017 and February 2018. Systematic sampling was conducted using patients’ files codes with a constant interval of five. Patients were included if they had a definitive diagnosis of AA or AGA made by an attending dermatologist based on clinical and histopathological findings. The Control group was selected from those healthy individuals referred to the dermatology clinics due to other irrelevant complaints such as skin spots. Exclusion criteria were other autoimmune diseases, lack of definitive diagnosis, being under treatment with anti-inflammatory drugs, or previously treated patients. The sample size was calculated using the findings of the study of Grace *et al*. [10]. According to the finding of this study, the mean frequency of mast cells using Giemsa staining was 15.14 ± 10.61 in patients with AA and 11.63 ± 2.97 in healthy controls. Considering the data mentioned above and a 95 percent confidence interval and 80 percent power, the sample size was calculated to be 20 subjects for each group. Demographic data of the study subjects, including age and sex, were recorded in the study-specific checklist. To investigate the inflammation and the presence of mast cells, 5 μm transverse slices were prepared from all paraffin-embedded and biopsy specimens fixed in formalin. All specimens were obtained from the scalp and prepared using Giemsa histochemically staining (Giemsa Staining Kit, Neutron Brand Made in Iran), which provides metachromatic staining of mast cell granules to accurately evaluate these cells [11]. With specific attention made to highlight the areas of greatest inflammation, perifollicular and perivascular areas were meticulously investigated. All slides from the specimens from three study groups including AGA, AA and healthy controls were evaluated separately by two dermatopathologists. By each sample, three high-power fields (×40) were evaluated and the number of perifollicular and perivascular mast cells was recorded. The dermatopathologists were blinded to the other dermatologist’s recordings and the study groups. The extent of the infiltration was divided into three categories: mild, moderate and severe according to the microscopic observations. Finally, the number of mast cells in the three study groups was compared in the perifollicular and perivascular regions.

###  Statistical Analysis

 The data were reported as mean ± standard deviation as well as minimum and maximum or frequency and percentage. Normal distribution of the variables was evaluated using the Kolmogorov–Smirnov test. For those normally distributed, one-way analysis of variance (ANOVA) was conducted to compare the data of the three groups. Tukey’s post hoc analysis was also conducted to determine the statistically significant differences in the mean number of mast cells in each group. For those variables without a normal distribution, the Kruskal–Wallis was used for comparing the data of the three groups. Then the Mann-Whitney-U test was conducted to compare two groups. Data were analyzed by SPSS software version 22 (SPSS Inc., Chicago). Correlation between variables was tested using Kendall’s Tau. For comparison of qualitative variables between two groups, the Fisher exact test or chi-square test was used. The numbers reporting the mast cells’ counts were rounded to make sense.

## Results

**Figure 1:**
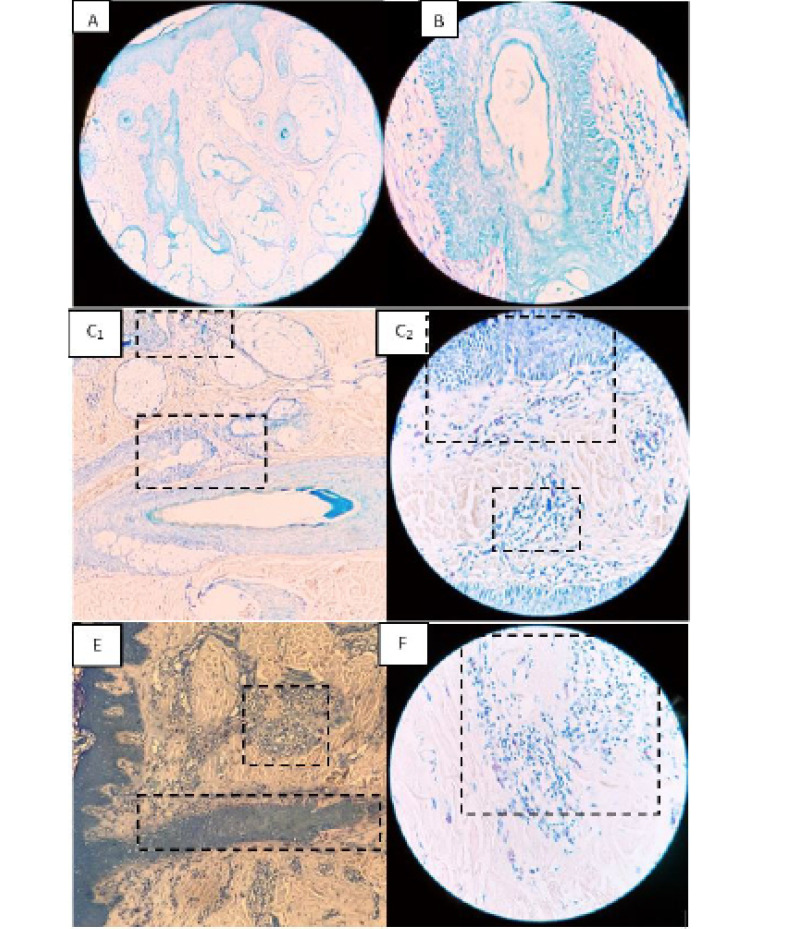
Mast cell presence in alopecia areata and androgenic alopecia in Giemsa staining, (black boxes indicate mast cells). (A) Control group specimen in low magnification (scanning magnification) (×4); (B) Control group specimen in high magnification (×40); (C) Alopecia areata specimen in low magnification (×4); (c) Alopecia areata specimen in high magnification (×40); (E) Androgenic alopecia specimen in low magnification (×4); (F) Androgenic alopecia specimen in high magnification (×40)

 Sixty specimens were evaluated from the included participants (20 subjects for each one of the three study groups). Demographic data demonstrated that no significant difference was seen among three groups in terms of sex; however, a significant age difference existed between groups (P=0.005, Table-1). Turkey’s post hoc analysis showed that there was not significant difference in age between subjects in AA (P=0.558) or AGA (P=0.065) groups and healthy controls. However, the AA group had a significantly lower age than the AGA group (P=0.004). Both the AA and AGA groups had significantly higher perifollicular (P=0.001 for both groups) and perivascular mast cell counts (P=0.001 and 0.005 for each group, respectively) (Figure-1). Although AA patients had similar perifollicular mast cell count with the AGA group (P=0.312), they had more frequent perivascular mast cells than the AGA group (P=0.042, Table-2).The data was further analyzed considering age (Table-3). Overall, 40, 10, and 30 percent of patients were over 40 years of age in AA, AGA, and control groups, respectively. Among patients aged under 40 years, perifollicular mast cell count was not significantly different between groups (P=0.086); however, subjects over 40 years of age in both groups had significantly higher perifollicular mast cell count than healthy participants (P=0.001 for both groups). A similar pattern of findings was noticed in perivascular mast cells. Although no significant difference was seen between perivascular mast cell count of AGA and control groups in patients under 40 years of age (P=0.142); significant differences were detected in perivascular mast cells between of AA and healthy groups in patients aged lower than 40 years (P=0.001) and also in those aged over 40 years (P=0.001). Also, patients over 40 years of age had higher perivascular mast cell counts in the AGA group than control (P=0.012). There was significant positive correlation between disease severity and mast cells counts in both perifollicular (correlation coefficient=0.671, P=.001) and perivascular areas (correlation coefficient=0.734, P=0.001) in AA patients. Moreover, in AGA patients, significant positive correlations were detected between disease severity and mast cells counts in perifollicular (correlation coefficient=0.701, P=0.001) but not in perivascular (correlation coefficient=0.178, P=0.349). Therefore, subgroup analysis was also conducted considering the severity of involvement in patients (Table-4). The proportions of patients with mild, moderate, and severe involvement were 30, 15, and 65 percent in the AA group and 25, 65, and 10 in the AGA group, respectively. Among mild cases, only patients with AA had a higher count of perifollicular mast cells than healthy group (P=0.015) and the perifollicular mast cells counts were not different between AA and AGA groups (P=0.752) and AGA and healthy control (P=0.153). However, significantly higher perivascular mast cell counts in both groups were seen in mild cases than healthy controls (P=0.001 for both comparisons).

## Discussion

Mast cells in mammalian skin are located in the dermis and subcutaneous tissue, especially in the connective tissue sheath of hair follicles and are involved in the hair folliclecycle [12-14]. A few studies have reported an increase in the number of mast cells in the perifollicular and perivascular in AA lesions [10,15,16]. Due to the importance of mast cells in non-scarring alopecias, the current study investigated the number of perifollicular and perivascular mast cells in AA and AGA lesions and compared them with healthy condition. According to the results of our study, the number of perifollicular and perivascular mast cells in both groups of AA and AGA patients were significantly higher than healthy controls. However, this association was partially age dependent as we detected that this difference was more prominent in those patients aged over 40 years. It should come with no surprise that no significant difference was observed in perifollicular and perivascular mast cell counts in AGA patients aged under 40 years compared to healthy control. This finding was consistent with previous studies reporting an age-related pattern of mast cell count [17-19]. To be more precise, it has been demonstrated that there is an increased number of mast cells in older ages [17]. We also found a severity based pattern in perifollicular and perivascular mast cell counts. Accordingly, our sub-group analysis revealed that in mild AGA cases there was no significant difference in perifollicular mast cell count compared to healthy control. However, the mast cell counts were significantly higher in moderate and severe AA and AGA cases than the healthy group. Grace *et al*. [10] also evaluated the follicular and non-follicular mast cell counts in patients with AA and AGA and reported similar mast cell counts with our results. Correspondingly, in their study, AA and AGA patients had significantly higher follicular and non-follicular mast cell counts than the healthy group. However, this study did not separately investigate the mast cell counts in perifollicular and perivascular areas. In furtherance, Cetin *et al*. [15] reported more frequent perifollicular and perivascular mast cell count in AA. However, they failed to detect a significant difference in perifollicular and perivascular mast cell count between mild and severe cases of AA. In an attempt to discover the role of mast cells in AA disease, Bertolini *et al*. revealed that there is an increased mast cell-CD8+ T-cell physical contacts as well as an augmented proliferation of perifollicular mast cells in AA patients [16]. They suggested that this interaction between mast cells and CD8+ T-cells may change the role of mast cells from an immune-inhibitory to a pro-inflammatory effect which leads to diminishing the immune privilege of the anagen hair follicles. This effect is mediated by upregulation of major histocompatibility complex class I molecules and downregulation of the expression of immune tolerance related molecules such as transforming growth factor beta-1 [16]. Accordingly, Zhang *et al*. confirmed that higher proliferation of mast cells in the perifollicular and perivascular areas of AA patients’ specimens positively correlated with CD8+ T lymphocytes count in perifollicular areas and negatively correlated with CD4+ T lymphocytes in perivascular areas [20]. Moreover, perivascular mast cells in AA are reported to be positively correlated with eosinophil presence [21]. These findings are all suggestive of the role of mast cells in the allergic and autoimmune process which precipitate the development of AA. Our study also detected that AA patients had higher perivascular mast cell count than AGA patients. Though, this difference was noticeable in patients aged over 40 years. Surprisingly, in mild cases, perivascular mast-cells were significantly less frequent in AA patients as compared with AGA patients. Similarly, in the study of Grace *et al*., mast cell count was higher in AA patients than that in AGA patients; however, this difference was not statistically significant which could be due to relatively small sample size (n=7 in AA and n=9 in AGA groups) or selecting patients from those aged under 40 or with different disease stages. Taken together, it is conceivable that mast cells are involved in the pathophysiology of both disease; however, the proliferation of mast cells in AA cases is more prominent. Therefore, along with the growing evidence that advocates the mast cell-related therapeutic strategies [22,23], our results suggest that these options may be also considered for patients with non-scaring alopecias particularly in AA. However, this idea should be investigated by future researches. Our study was among the few studies that evaluated the presence of mast cells in the AA and AGA patients’ specimens. Moreover, in our study, the association of mast cells counts in these diseases was investigated with respect to age and the severity of diseases. However, our study had some limitations that should be described. Due to the relatively small sample size, the number of patients in subgroup analysis was low. Moreover, few male patients were included in our study that prevented us from performing subgroup analysis in male and female patients individually. Furthermore, our study did not evaluate the pathophysiology underlying the higher proliferation of mast cells in AA and AGA patients. Therefore, this subject can be an interesting topic for future studies.

## Conclusion

 Based on the result of our study, there is a significant increase in proliferation and infiltration of mast cells in AA and AGA patients. However, the increase pattern is different between those aged under 40 years and those aged over 40 years. In particular, the increase in mast cell infiltration is more prominent in those patients aged over 40 years. Moreover, there is a significant relationship between mast cell infiltration and the severity of the disease, supporting their role in the progression of the disease. Moreover, an increase in mast cell proliferation is more dominant in AA patients than AGA patients.

## Acknowledgement

 None

## Conflict of Interest

 None declared.

**Table 1:** Demographic Data of Patients Included in Three Groups

**Parameters**	AGA	AA	Control	P-value
**Age mean ± SD (min-max)**		51.30 ± 6.90 (37 - 64) †	43.75 ± 7.44 (29 - 57) †	46.10 ± 7.18 (35 - 58)	0.005*
**Sex n (%)**	Female	19 (95)	16 (80)	17 (85)	0.364**
	Male	1 (5)	4 (20)	3 (15)	

* One-way ANOVA test was used. ** Fisher’s exact test was used. Turkey’s post hoc analysis showed no significant difference between AGA and AA groups in comparison with healthy controls. † Significant difference between AA and AGA **AA: **Alopecia Areata, AGA: Androgenic Alopecia

**Table 2:** Mast Cell Counts in Specimens of Subjects in the Three Groups

**Mast cells count**	**AGA**	**AA**	**Control**	**P-value**
**Perifollicular mean ± SD ** **( min -max)**	18 ± 10 (5 - 47) ^**^	23 ± 12 (8 - 54)**^**^	5 ± 4 (0 - 16)	0.001*
**Perivascular mean ± SD ** **( min -max)**	9 ± 5 (4 – 19)**^**^†	14 ± 10 (4 - 42)**^**^†	3 ± 2 (1 – 6)	0.001*

* Kaushal-Wallis test was used. ** Significant difference in comparison with healthy control by Mann-Whitney U test † Significant difference between AA and AGA in Mann-Whitney U test AA: Alopecia Areata, AGA: Androgenic Alopecia

**Table 3:** Mast Cell Counts in Specimens of Subjects in the Three Groups Considering the Age

**Mast cells count**	**Age (year)**	**AGA**	**AA**	**Control**	**P-value**
**Perifollicular mean ± SD ** **( min -max)**	<40	13 ± 4 (9 - 17)	20 ± 15 (8 - 54)	5 ± 2 (0 - 16)	0.086
	>40	19 ± 11 (5 - 47) ^**^	25 ± 10 (12 - 41)**^**^	6 ± 4 (0 - 16)	0.001*
**Perivascular mean ± SD ** **( min -max)**	<40	7 ± 1 (6 – 8)	9 ± 5 (4 - 18)**^**^	3 ± 2 (1 – 5)	0.041*
	>40	10 ± 5 (4 – 19)**^**^†	18 ± 10 (6 - 42)**^**^†	3 ± 2 (1 – 6)	0.001*

* Kaushal-Wallis test was used. ** Significant difference in comparison with healthy control by Mann-Whitney U test † Significant difference between AA and AGA in Mann-Whitney U test **AA:** Alopecia Areata, AGA: Androgenic Alopecia

**Table 4:** Mast Cell Counts in Specimens of Subjects in the Three Groups Considering Involvement Severity

**Mast cells count**	**Involvement**	**AGA**	**AA**	**Control††**	**P-value**
**Perifollicular mean ± SD ** **( min -max)**	Mild	9 ± 4 (5 - 16)	11 ± 3 (8 - 15)**^**^	5 ± 4 (2 - 8)	0.011
	Moderate	19 ± 6 (10 - 31) ^**^	21 ± 3 (18 - 23)**^**^	5 ± 4 (2 - 8)	0.001*
	Severe	41 ± 8 (35 - 47) ^**^	30 ± 11 (17 - 54)^**^	5 ± 4 (2 - 8)	0.001*
**Perivascular mean ± SD ** **( min -max)**	Mild	8 ± 1 (6 – 10)**^**^†	6 ± 1 (4 - 7)**^**^†	3 ± 2 (1 – 6)	0.001*
	Moderate	8 ± 5 (4 – 19)**^**^	10 ± 3 (8 - 14)**^**^	3 ± 2 (1 – 6)	0.001*
	Severe	18 ± 1 (17 – 19)**^**^	20 ± 9 (8 - 42)**^**^	3 ± 2 (1 – 6)	0.001*

* Kaushal-Wallis test was used. ** Significant difference in comparison with healthy control by Mann-Whitney U test † Significant difference between AA and AGA in Mann-Whitney U test †† Control group data was considered in total when comparing the AA and AGA groups in three levels of disease severity **AA: **Alopecia Areata, AGA: Androgenic Alopecia
